# Editorial for Special Issue “Ophthalmology: New Diagnostic and Treatment Approaches”

**DOI:** 10.3390/medicina61091539

**Published:** 2025-08-27

**Authors:** Stephen G. Schwartz, Krishna S. Kishor, Victor M. Villegas, Christopher T. Leffler, Andrzej Grzybowski

**Affiliations:** 1 Department of Ophthalmology, Bascom Palmer Eye Institute, University of Miami Miller School of Medicine, Naples, FL 34103, USA; 2 Department of Ophthalmology, Bascom Palmer Eye Institute, University of Miami Miller School of Medicine, Palm Beach Gardens, FL 33418, USA; kkishor@med.miami.edu; 3 Department of Ophthalmology, University of Puerto Rico School of Medicine, San Juan, PR 00936, USA; victor.villegas@upr.edu; 4 Department of Ophthalmology, Virginia Commonwealth University School of Medicine, Richmond, VA 23298, USA; chrislefflermd@gmail.com; 5 Foundation for Ophthalmology Development, 61-836 Poznan, Poland; ae.grzybowski@gmail.com

## Introduction

There have been many recent advances in the diagnosis and treatment of eye diseases. The aim of this Special Issue is to publish new evidence and to review the current state-of-the-art developments in the diagnosis and treatment of vision-threatening diseases. This Special Issue features nine original contributions, including basic science investigations, clinical studies, and review articles.

Ichikawa and colleagues [1] investigated fibronectin adhesion to three types of intraocular lenses (IOLs): collamer-based EVO + Visian implantable contact lenses (ICLs); hydrophilic acrylic implantable phakic contact lens (IPCLs); and hydrophilic acrylic phakic-IOLs (LENTIS) used as controls. Under the experimental conditions studied, minimal fibronectin deposition and cellular adhesion was noted in the central optical zone of all three IOLs. However, the haptics of the collamer IOLs developed a thin fibronectin film. The investigators concluded that the collamer IOLs may promote fibronectin film formation, which may affect long-term transparency and biocompatibility.

Bartusis and colleagues [2] conducted a cross-sectional observational study comparing intracranial pulse wave signals in patients with normal-tension glaucoma (NTG) and age-matched controls. The investigators reported that median intracranial pulse wave amplitude was significantly higher in patients with NTG, which suggests that this noninvasive screening device may serve as a potential biomarker for this difficult-to-diagnose condition.

Sicks and colleagues [3] irradiated porcine and human corneas using 222 nm Far-UVC and reported that treatment with up to 60 mJ/cm^2^ did not significantly affect corneal cell integrity and caused only a 3.7% reduction in cell density. The investigators proposed that irradiation under these conditions may be effective in decontaminating donor corneas, thus increasing the available supply of tissue for surgical patients.

Otake and colleagues [4] studied the effects of increasing ambient temperature on human lens epithelial cells. Using shotgun liquid chromatography with tandem mass spectrometry-based global proteomics, they reported differences in proteins digested from the immortalized human lens epithelial cell line iHLEC-NY2 at normal (35 degrees C) and warming (37.5 degrees C) temperatures. They proposed that differences in protein expression under warming conditions may contribute to the pathogenesis of cataract development.

Chen and colleagues [5] applied Shapley additive explanation (SHAP) techniques with machine learning to review intraocular pressure changes among pediatric patients treated with atropine to slow myopia progression. The investigators proposed that this approach may improve our ability to make more personalized treatment decisions for these patients.

Murati Calderón and colleagues [6] reviewed emerging therapies for retinitis pigmentosa. Voretigene neparvovec is approved for the treatment of patients with *RPE65*-associated disease. Gene-based therapeutic strategies currently being investigated include gene replacement therapies, gene editing using CRISPR-Cas9, RNA-based therapies, and optogenetic therapies.

Ruiz-Justiz and colleagues [7] presented current practices for the surgical care of pediatric patients requiring combined cataract surgery and pars plana vitrectomy. This approach is generally used for patients with congenital cataracts, ectopia lentis, retinopathy of prematurity, retinal detachment, and persistent fetal vasculature. The advantages and disadvantages of lens-sparing vitrectomy versus combined surgery are discussed.

Rosado and colleagues [8] surveyed the pathogenesis and management of Bardet-Biedl syndrome. Clinical manifestations include retinal degeneration, corneal abnormalities, strabismus, nystagmus, cataracts, and optic nerve abnormalities. Investigational therapies include gene replacement therapies, gene-editing using CRISPR-Cas9, nonsense suppression (readthrough) therapy, and neuroprotective therapies.

Olawade and colleagues [9] reviewed applications of artificial intelligence to ophthalmology, including machine learning and deep learning techniques. The investigators report that artificial intelligence may improve diagnostic accuracy and personalized treatment plans for patients with ophthalmic diseases.

We believe that these nine contributions improve our current understanding of ophthalmology and have the potential to stimulate further research.
